# Neuromyelitis Optica Spectrum Disorder and Autoimmune Rheumatological Diseases: A Report of Two Cases and Literature Review

**DOI:** 10.7759/cureus.26138

**Published:** 2022-06-20

**Authors:** Mohannad Faisal, Ahmad Matarneh, Israa Alshahwani, Omar Al-Allaf, Abdul-wahab Al-Allaf

**Affiliations:** 1 Internal Medicine, Hamad Medical Corporation, Doha, QAT; 2 General Practice, Hamad Medical Corporation, Doha, QAT; 3 Rheumatology, New Cross Hospital, Wolverhampton, GBR; 4 Rheumatology, Hamad Medical Corporation, Doha, QAT

**Keywords:** nmosd, sjogren’s syndrome, rheumatology & autoimmune diseases, devic's disease, behcet’s syndrome, neuromyelitis optica spectrum disorder

## Abstract

Neuromyelitis optica spectrum disorder (NMOSD) is a rare autoimmune disorder, and its association with other autoinflammatory diseases has been reported in this study. In this article, we are discussing two patients with neuromyelitis optica who show evidence of autoimmune rheumatic diseases. The first case presented with acute myelitis and was diagnosed with NMOSD; she developed clinical features of Behçet’s disease during follow-up, making it the second reported case worldwide. The second case presented with neuromyelitis optica and was found to have strongly positive Sjogren’s serology.

## Introduction

Neuromyelitis optica spectrum disorder (NMOSD) is an autoimmune inflammatory disorder of the central nervous system (CNS). It is characterized by inflammation and demyelination of the optic nerve and the spinal cord, in addition to other parts of the CNS. It is associated with a high serum aquaporin-4 immunoglobulin-G antibody (AQP4-IgG), which is highly specific for the disease and may play a role in its pathogenesis [1]. Diagnosis of NMOSD needs the presence of one of the core clinical characteristics in addition to the positivity of AQP4-IgG. If the AQP4 antibody is negative, more strict criteria are applied. The core clinical characteristics include one of six CNS regions: optic nerve, spinal cord, area postrema of the dorsal medulla, brainstem, diencephalon, or cerebrum [2]. Neuroimaging is a key in the diagnosis of NMOSD and in the differentiation of it from other demyelinating diseases, including multiple sclerosis (MS). Magnetic resonance imaging (MRI) of the brain and spine typically shows the detection of a longitudinally extensive myelitis lesion in the spinal cord associated with acute myelitis, which is the most specific neuroimaging characteristic of NMOSD and is rare in MS [3].

Behçet’s disease (BD) is also a systemic immune-inflammatory disorder, which is characterized clinically by recurrent multiple oral and genital ulcerations, uveitis, and other skin manifestations. It is more common among people who lived along the ancient Silk Road. Its diagnosis is clinical and depends on the criteria derived from a study of 914 patients from 12 centers in seven countries (International Study Group for Behçet's Disease, 1990). The agreed diagnostic criteria require the presence of recurrent oral ulceration (three times in one year) plus two of the following: recurrent genital ulceration, eye lesions (uveitis or retinal vasculitis), skin lesions (erythema nodosum, pseudo-folliculitis, papulopustular lesions, or acneiform nodules), or a positive pathergy test [4]. The cause of BD is unknown, but an autoimmune reaction triggered by an infectious or environmental agent (possibly local to a geographic region) in a genetically predisposed individual seems most likely [5].

Sjogren’s syndrome is another autoimmune inflammatory disorder characterized by lymphocytic infiltration of typically the salivary and lacrimal glands. It can be either primary or secondary to other autoimmune diseases. Symptoms may be attributed to exocrine or non-exocrine diseases. The exocrine disease leads to dryness of the eyes, upper aerodigestive tract, vagina, and skin and may also involve the liver, pancreas, hepatobiliary system, gastrointestinal tract, and kidneys. Non-exocrine diseases include vasculitides, hematological disorders, and thyroiditis. Diagnosis of Sjogren's syndrome requires typical clinical symptoms and signs supported by serological tests [6].

## Case presentation

First case

A 40-year-old Arabic Asian lady with a history of hypothyroidism presented in August 2019 with right-sided body weakness for the previous few days. It was preceded by the neck, right arm, and shoulder pain with numbness on the right side of her body for one month. She reported some blurring of vision in both eyes along with episodes of urinary retention. She vomited a few times before admission, but she had no fever or headache. The patient has had no similar or other neurological symptoms before. She denied previous trauma; she is a non-smoker with no alcohol consumption, and she did not have any specific dietary restrictions. The examination shows stable vital signs with normal mental status and normal cranial nerve examination. She has a weakness of grade 4/5 on the right side of her body with hyperreflexia and a positive Babinski and Hoffman sign on both sides. She also has diminished sensation, which is more on the right side of her body. Other systemic examinations were unremarkable.

The patient's laboratory investigation showed normal serum chemistry with high vitamin B12 (vit-B12), mild anemia, and a negative autoimmune screen. Cerebrospinal fluid analysis showed lymphocytic pleocytosis, and infectious causes were excluded.

**Figure 1:**
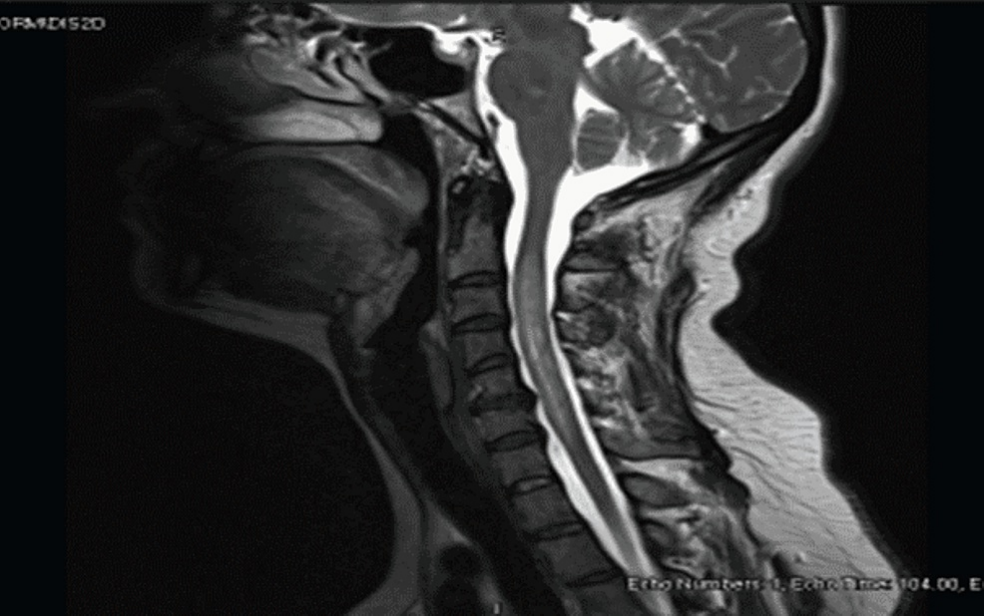
MRI of the cervical spine A long segment of heterogeneously bright T2 signal intensity in the cervical spinal cord extending from C2 down to C7 vertebral level with mild cord expansion. MRI: Magnetic resonance imaging.

Patient MRI of the head and cervical spine (Figure 1) showed a long segment of heterogeneously bright T2 signal intensity in the cervical spinal cord extending from C2 down to C7 vertebral level with mild cord expansion. Incomplete peripheral enhancement is noted in the postcontrast series. The changes are mainly involving the peripheral white matter component of the spinal cord. No displacement of the central spinal canal with relative sparing of the gray matter was noted. The most probable radiological diagnosis was neuromyelitis optica.

The patient was given intravenous methylprednisolone of 1 g daily for five days, and she showed significant improvement. She has been discharged on a reducing dose of 40 mg of oral prednisolone with neurology outpatient follow-up. Her serum aquaporin-4 receptor antibody came back positive, and the patient was diagnosed with NMOSD. It was planned to start her on rituximab, which was postponed as the patient was found to have latent tuberculosis that is common in our area. She was kept on a reducing dose of oral steroids while finishing her treatment for latent tuberculosis, which was then stopped.

The patient was readmitted a month later with a paroxysmal tonic spasm affecting all limbs and was diagnosed with a flare of NMOSD. She was given another course of intravenous methylprednisolone. Because of the severity of her symptoms, a decision was made to start her on methylprednisolone 1 g for five days, which has been administered. For paroxysmal tonic spasms, multiple regimens of antiepileptic and anti-spasmodic were tried until her symptoms were controlled on lacosamide, oxcarbazepine, and baclofen.

**Figure 2:**
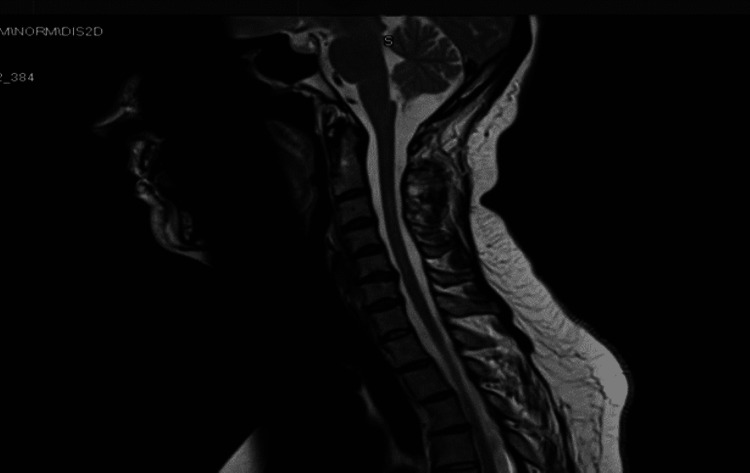
MRI of the cervical spine showing the reduction in T2 hyperintensity of intramedullary abnormal signal intensity extending from C2 to C7 level MRI: Magnetic resonance imaging.

The patient was given her first rituximab course, which consists of two one-gram doses two weeks apart, which has been repeated six months later and was planned to be repeated once every six months depending on her condition and the lymphocyte subset counts. Upon a 15-month follow-up, she showed a significant improvement in her symptoms, and her repeat MRI spine showed a reduction in the T2 hyperintensity of the previous intramedullary abnormal signal intensity extending between C2 and C7 levels with no evidence of abnormal postcontrast enhancement or new lesions (Figure 2).

Meanwhile, the patient was referred to a rheumatology clinic in May 2021 for having recurrent oral ulceration that had been reported whenever her prednisolone dose was tapered below 10 mg and disappeared when the dose of steroid was increased. The ulcers were painful and were associated with recurrent genital ulcers. She also reported skin rashes. The patient denied having joint pain or vision problems. She has been diagnosed with Behçet's disease and was started on colchicine 500 μg three times daily with a short course of oral steroids. Human leukocyte antigen (HLA)-B51 was negative, and a detailed eye examination by ophthalmology revealed no eye involvement.

Upon follow-up four months later, the oral symptoms were much better, but the ulcers recurred if the prednisolone dose was tapered below 10 mg, so a steroid-sparing agent was tried. The patient was maintained on mycophenolate mofetil (MMF), which should cover both diagnoses, and her colchicine dose has been increased to 1 mg bid.

The last follow-up with rheumatology and neurology in early 2022 showed good control of her symptoms both from an NMOSD and Behçet's disease point of view, with a plan to keep her on colchicine, MMF, and the least effective dose of steroids for BD and the rituximab to be used once every six months. She is doing very well with this regimen without any further flares of her NMOSD. Currently, the patient is maintained on rituximab courses guided by the lymphocyte subclasses with the aim of increasing the duration between rituximab doses to prevent further NMOSD relapses. Our future target for the patient is to be maintained only on mycophenolate along with the colchicine.

Second case

A 32-year-old female, previously healthy, presented to the hospital with a two-week history of back pain. The pain was mostly in the middle of the back, with radiation to the lower back. She also reported a one-week history of gradual bilateral lower limb weakness, limiting her ability to walk. This was associated with perianal numbness and difficulty passing urine and stool. She had no headache, fever, or abnormal body movements. She has had no previous neurological symptoms. She denied viral or diarrheal illness in the preceding month. She also denied having sicca-like symptoms, joint pain, or skin rashes.

On presentation, she was vitally stable with normal mental status. She had an unremarkable cranial nerve examination. Upper limb motor and sensory examinations were normal. Neurological examination of the lower limbs revealed weakness mostly around the hips and knees with diminished sensation. Her ankle reflexes were absent bilaterally. She had saddle anesthesia and diminished anal tone typical of Cauda equina lesion. Examination of other systems was unremarkable.

Serum chemistries and complete blood counts were unrevealing. Contrast MRI of the head and spine revealed extensive leptomeningeal enhancement involving the whole spinal cord with a central cord hyperintensity between T10 and the Cauda equina. Cerebrospinal fluid (CSF) analysis revealed a protein of 1.7 g/L and glucose of 2.35 mmol/L. White blood cells in CSF were 89/μl predominantly lymphocytic (99%). Antinuclear antibody was positive at 1/80 with strongly positive anti-Sjogren’s syndrome antigen A (anti-Ro/SSA) and Sjogren’s syndrome antigen B (La/SSB) antibodies.

During her hospital stay, her lower limb weakness worsened, and she developed urine retention. Thus, intravenous methylprednisolone, 1 g per day, was given for five days with subsequent improvement in her clinical condition. Upon follow-up, the anti-aquaporin-4 antibody (AQP4-Ab) came strongly positive with a titer of 1:10,000, thus confirming the diagnosis as NMOSD. Two doses of one-gram rituximab were added to her treatment two weeks apart. Then, she was maintained on rituximab courses roughly every six months as guided by her symptoms and the lymphocyte subclasses levels to prevent further NMOSD relapses.

## Discussion

The association between autoimmune diseases is common. The combination of at least three autoimmune diseases in the same patient has been defined as multiple autoimmune syndromes (MAS). Approximately, 25% of patients with autoimmune diseases develop new autoimmune diseases [7]. Familial, genetic, infectious, immunologic, and psychological factors have been implicated in the development of MAS [8,9]. This association sometimes causes challenges in the management of these patients and warrants monitoring and surveillance of patients who develop one autoimmune disease for the development of another [10].

Some of the autoimmune diseases are commonly associated with other specific autoimmune diseases such as the common association of Sjogren's syndrome with systemic lupus erythematosus, systemic sclerosis, or rheumatoid arthritis [11], while other autoimmune diseases are rarely associated with each other. Although NMOSD is an idiopathic autoimmune inflammatory disease of the CNS, some association with other autoimmune diseases like Sjogren’s syndrome has been proposed [12].

Our first patient was diagnosed with neuromyelitis optica after presenting with acute neurological illness, typical MRI changes, and positive anti-(AQP4-IgG) antibody. Upon two years of follow-up, she developed recurrent painful oral and genital ulcerations and received a clinical diagnosis of BD. To the best of our knowledge, the association of NMOSD with BD has only been reported once in a case when a 21-year-old male patient who presented with recurrent arthritis and oro-genital ulceration in addition to lower limb weakness was found to have a long segment of demyelination in the cervical spinal cord and optic nerve on MRI. However, in contrast to our case, interestingly, that case was found to have negative anti-(AQP4-IgG) antibody [13]. We do not have a clear explanation for the association due to the rarity of the data. However, one study found enhanced complement activation in both NMOSD and BD in comparison to healthy controls and MS patients [14]. Further research is needed to identify the biological basis for such an association, which will aid in the better understanding and management of these diseases.

Our second patient was a case of NMOSD who was diagnosed based on the presence of longitudinal myelitis (LM) and AQP4-Ab. Interestingly, she also had positive anti-SSA/SSB, but its significance was debated in the absence of other suggestive clinical signs and symptoms of Sjogren’s syndrome. A literature review showed that positive anti-SSA/SSB and other autoimmune antibodies could be present in patients with NMOSD. An elevated humoral response can explain this seropositivity [15], or both conditions could coexist. In one study, 18 (37%) out of 48 patients with AQP4-Ab-positive NMOSD tested positive for anti-Ro/SSA [16]. In another study, most Sjogren's syndrome patients presenting with LM are AQP4-Ab positive [17]. There are some overlaps between NMOSD and the primary Sjogren’s syndrome [18], and this possibly points out that something is common in the pathogenesis of these conditions. Special attention needs to be considered for a patient with Sjogren’s syndrome who presents with neurological symptoms as this may point to the diagnosis of NMOSD, which will change the management plan and prognosis [19].

Another learning point from our second case is that for patients presenting with Cauda equina symptoms, medical cases such as NMOD should be entertained as the management is different from all other causes of Cauda equina. As compared to other rheumatological diseases that can be treated with rituximab, such as rheumatoid arthritis, we must be very vigilant and have a very low threshold to repeat the courses of rituximab in NMOSD. This is because each attack of an NMOSD flare could result in residual neurological damage, which we need to avoid.

## Conclusions

NMOSD is a rare disease and its association with other autoimmune diseases needs to be highlighted. Physicians should have a high index of suspicion for considering NMOSD in any patient with Sjogren’s syndrome presenting with neurological symptoms as therapeutic options and prognosis may differ accordingly, while the NMOSD and BD associations need further studies to be established. In patients with Cauda equina, medical causes including NMOSD should be considered.
